# Optimal conditions for quantification of IRF4 mRNA and protein as a correlative biomarker in clinical cancer studies

**DOI:** 10.1186/s12967-026-07803-0

**Published:** 2026-03-16

**Authors:** Joshua D. Hartman, Silvia Vicenzi, Alexander A. Tong, Lara C. Avsharian, Ashni A. Vora, Caitlin Costello, Ida Deichaite, Leslie A. Crews

**Affiliations:** 1 Division of Regenerative Medicine, Department of Medicine; University of California San Diego, La Jolla, CA 92093 USA; 2 Moores Cancer Center, University of California San Diego, La Jolla, CA 92093 USA; 3 Sanford Stem Cell Institute, University of California San Diego, La Jolla, CA 92037 USA; 4 Division of Blood and Marrow Transplantation, Department of Medicine, University of California San Diego, La Jolla, CA 92093 USA; 5 Department of Radiation Medicine and Applied Sciences, University of California San Diego, La Jolla, CA 92093 USA; 6 Present Address: Irell & Manella Graduate School of Biological Sciences, City of Hope, Duarte, CA 91010 USA; 7 Present Address: Laboratory of Lymphocyte Dynamics, The Rockefeller University, New York, NY 10065 USA

**Keywords:** Processing variables, Molecular biomarkers, Peripheral blood, Myeloma, IRF4

## Abstract

**Background:**

The transcription factor interferon-regulatory factor-4 (IRF4) has recently been highlighted as a valuable biomarker due to its common dysregulation and association with poor prognoses in hematological malignancies such as multiple myeloma (MM). With several clinical programs for the inhibition of IRF4 currently in development, determination of the optimal sample collection conditions and quantification methods will facilitate optimal selection of patients for these treatments, along with enabling robust monitoring of their molecular responses to IRF4 inhibitory agents over time.

**Methods:**

Using peripheral blood samples from healthy donors, we evaluated IRF4 mRNA and protein stability across a range of pre-analytical conditions including blood collection tube type (comparing EDTA, Paxgene ccfDNA, and Na Citrate), sample processing interval (24 to 48 hours after sample collection), and cell population isolated (total mononuclear cells, CD3^+^ T lymphocytes, CD14^+^ monocytes, and CD19^+^ B lymphocytes). We sought to further determine the extent to which post-processing variables such as cell yield, RNA yield, and housekeeping gene (*ACTB* versus *HSPA5* and *ATF4*) selection may influence measurements of IRF4 mRNA and protein abundance. RNA and protein quantification endpoints included qRT-PCR and intranuclear flow cytometric analyses, respectively, to characterize the cell surface and intracellular molecular signatures.

**Results:**

In qRT-PCR biomarker studies, we found that human IRF4 expression was detectable in all cell fractions (PBMCs, CD19^+^ B cells and CD3^+^ T cells) evaluated and tube types tested (Na Citrate, EDTA, Paxgene ccfDNA). However, transcript stability of hIRF4 and the well-established reference gene β-actin (*ACTB*), as inferred from raw quantification cycle (Cq) values, varied substantially across tube types and between processing delay time points. mRNA levels of IRF4 along with an alternate, MM-optimized, reference gene, *HSPA5*, were determined to exhibit optimal stability in samples collected in Na Citrate preservative tubes. This tube type also offered the highest stability of immune cell surface antigens across 24–48-hour processing delays. Intranuclear IRF4 protein levels were remarkably stable in all cell populations tested across all tube types, with the highest relative expression in CD14^+^ and CD19^+^ fractions, and lowest in CD3^+^ fractions, in this cohort of healthy donors.

**Conclusions:**

Overall, Na Citrate tubes maintained the stability of IRF4 mRNA and protein expression even after sample processing intervals of up to 48 hours post-collection. Furthermore, IRF4 protein appears to be slightly more stable than mRNA after variable processing delays, but both analytes are expected to be detectable and relatively stable in clinical samples, thus enabling reliable analysis of IRF4 as a molecular biomarker in clinical samples collected from multiple sites and shipped to a central laboratory.

**Supplementary information:**

The online version contains supplementary material available at 10.1186/s12967-026-07803-0.

## Background

As we enter an era of precision medicine, cancer research is poised to advance dramatically as laboratory and clinical investigators begin to integrate RNA and protein indicators of tumor cell abundance and function into clinical decision-making and care. With these advances already underway, it is essential to identify optimal sample collection, shipment, and analysis pipelines that will enable reliable quantification of candidate diagnostic and prognostic biomarkers [1, 2]. Among these biomarkers, the transcription factor interferon-regulatory factor-4 (IRF4) has recently been highlighted as a valuable biomarker due to its common dysregulation and association with poor prognoses in myeloid and lymphoid neoplasms alike [3–5]. Furthermore, under physiological conditions IRF4 mediates immune responses, however it acts as an oncogene when irregularly expressed in lymphoplasmacytic neoplasms [6]. We previously completed comprehensive studies of an antisense oligonucleotide (ASO) agent targeting IRF4 in pre-clinical models of high-risk multiple myeloma (MM), an aggressive plasma cell neoplasm [5]. In addition, we established sensitive quantitative protein detection protocols to detect IRF4 protein in human cells after in vivo treatment with IRF4 ASOs [4]. With clinical programs for this agent, also known as frenlosirsen (ION251, NCT04398485), and other indirect IRF4-targeted compounds (NCT06433947), in development, we sought to determine the optimal sample processing and analysis conditions that would enable accurate quantification of immune biomarker mRNA and protein in immune cells to inform planned molecular biomarker studies for IRF4 modulator therapies.

We tested several pre- and post-processing variables and pre-analytical variables that we anticipated were the most essential to guiding clinical trial correlative study design for immune cell biomarkers like IRF4 [7, 8]. First, using peripheral blood samples from healthy donors, we evaluated IRF4 mRNA and protein stability across a range of pre-analytical conditions including blood collection tube type (comparing EDTA, Paxgene ccfDNA, and Na Citrate), sample processing interval (24 to 48 hours), and cell population isolated (total mononuclear cells, CD3^+^ T lymphocytes, CD14^+^ monocytes, and CD19^+^ B lymphocytes). Building upon prior studies that established a specific set of reference genes (*HSPA5*, *ATF4*, and *UBA52*) exhibiting optimal stability for qPCR-based biomarker studies for MM clinical samples [9], we sought to further determine the extent to which post-processing variables such as cell yield, RNA yield, and housekeeping gene (*ACTB* versus *HSPA5* and *ATF4*) selection may influence measurements of IRF4 mRNA and protein abundance. RNA and protein quantification endpoints included qRT-PCR and intranuclear flow cytometric analyses, respectively, to characterize the cell surface and intracellular molecular signatures.

## Methods

### Sample acquisition and study design

For sample collection and analysis optimization, whole blood samples from a total of 5 non-smoking donors were obtained commercially from AllCells (Table 1). The blood specimens were shipped in 2 shipments to optimize logistics during sample collection (donor scheduling, etc.), with shipments including 2–3 samples at a time. For each donor, 2 specimens of 3 different tube types were obtained: Paxgene ccfDNA (8.5 mL), Na Citrate (4.5 mL) and EDTA (10 mL). One of each tube type for each donor was processed immediately upon receipt (24hrs after collection), while the second of each tube type was stored at room temperature on a rocker set on low speed to mimic shipping conditions. The second tube of each type was then processed the next day (24hrs after receipt, which represented 48hrs after collection). The times of blood draw/collection and processing were recorded to ensure each sample was processed immediately after the planned experimental delay interval. Cells were counted and viability assessed using trypan blue staining (1:1 dilution) according to standard protocols to determine the number of live cells per milliliter.

**Table 1:**
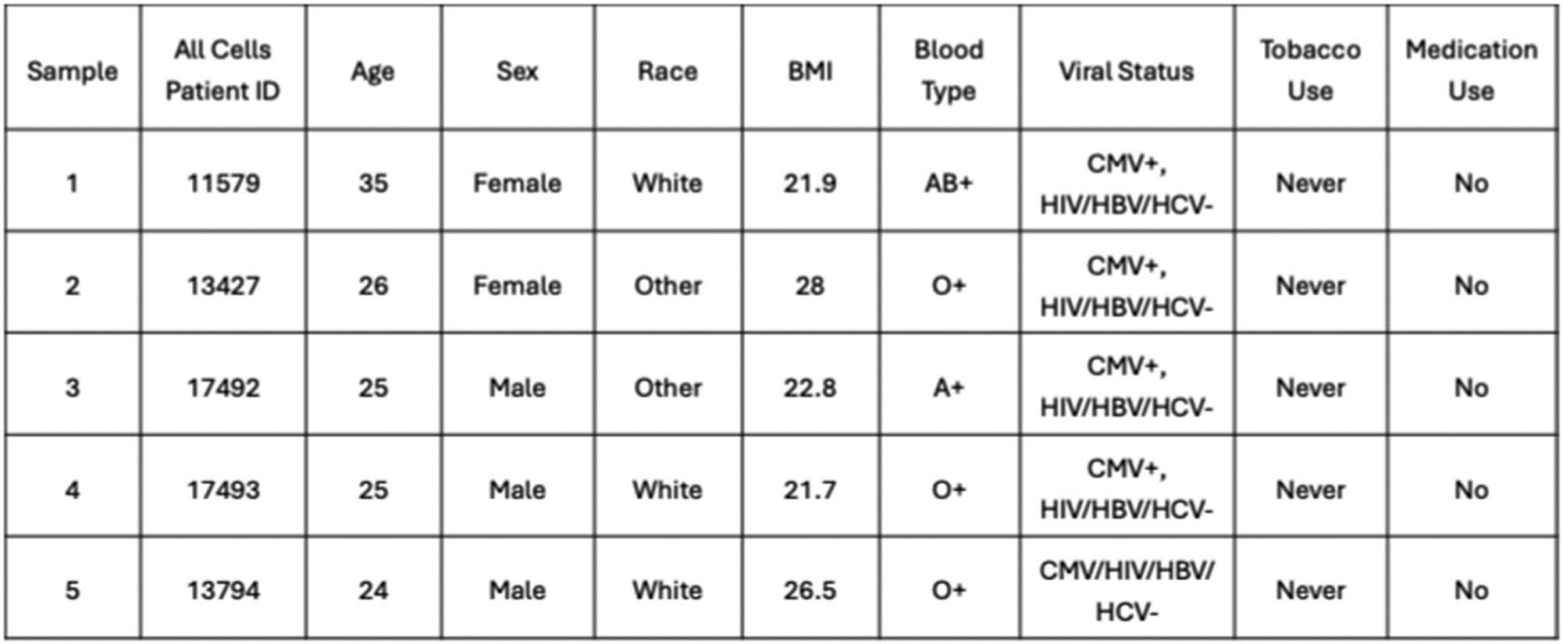
Summary of peripheral blood sample donor demographics and attributes (healthy donors included in the optimization cohort

### PBMC isolation and cell fraction selection

**Fig. 1:**
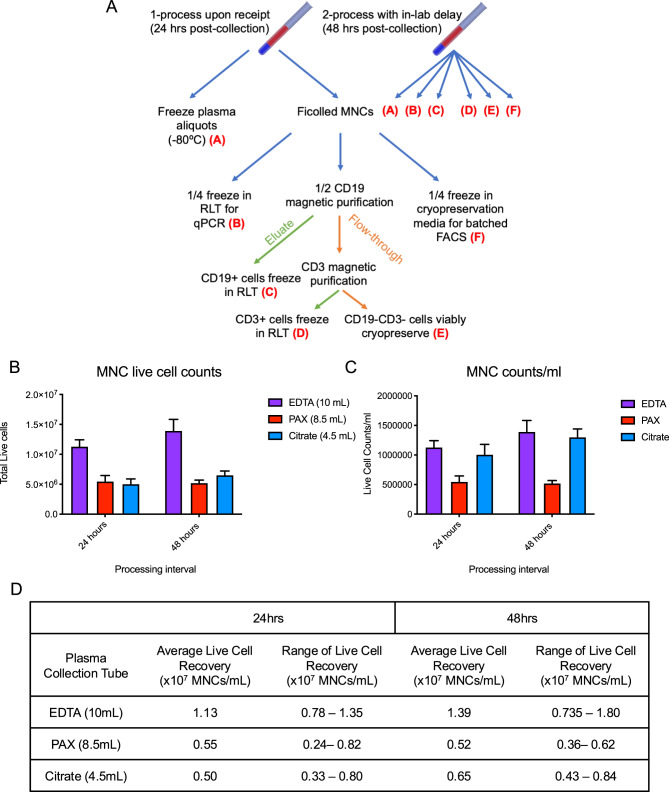
Study overview and cell viability measurements. (**A**) Schematic diagram of the study design and workflow for sample processing and analysis after receipt of each peripheral blood sample. For each timepoint (24 and 48 hrs post-collection), one tube of each preservative type was collected for each healthy donor sample (*n* = 5 individual donors). (**B**, **C**) Bar graphs showing total live cell counts and tube volumes (**B**) and live cell counts per mL (adjusted to total tube volume) (**C**) of each tube type (EDTA, Na Citrate, PAXgene) for *n* = 5 individual donors, grouped by 24- and 48-hr processing intervals. (**D**) Summary of cell recovery averages and ranges (based on data from panel **B**) across each experimental condition (*n* = 5 donors)

All samples were subjected to plasma separation followed by mononuclear cell isolation by Ficoll density gradient centrifugation. First, plasma fractions were isolated by centrifugation at 200 ×g for 10 minutes at room temperature, with the braking level set to medium. Up to one half of the estimated plasma volume (supernatant) was removed and snap frozen for use as control plasma samples in other studies. An equivalent volume of HBSS (without calcium or magnesium) was supplemented back to the blood tube. The Ficoll-Paque (GE Healthcare) standard density gradient centrifugation protocol was completed as described by the manufacturer. Afterward, a fraction of each MNC sample was cryopreserved and the remainder of the sample was further subjected to magnetic purification of B (CD19+) and T (CD3+) cells to further isolate immune cell subpopulations (Fig. 1A). Purification was carried out via magnetic microbead isolation (Miltenyi) using LS selection columns (Miltenyi) and CD19 and CD3 microbeads (Miltenyi), according to the manufacturer’s instructions. qRT-PCR analyses were performed for *hIRF4*, *ACTB*, *HSPA5*, and *ATF4* for total MNC, CD19+ and CD3+ fractions (30 samples collected per fraction, see Fig. 1A).

### RNA extraction and cDNA preparation

Once the PBMCs and immune cell fractions (CD3, CD19) were isolated, total RNA was extracted from each individual sample using the AllPrep DNA/RNA Mini Kit (Qiagen) and RNeasy Mini Spin Columns (Qiagen) according to the manufacturer’s instructions. Additional aliquots of culture human MM cell lines (RPMI-8226, ATCC) were also processed as positive control samples for IRF4 expression. RNA concentrations were measured via a NanoDrop One Spectrophotometer (ThermoFisher). The RNA samples were stored at −80 °C until subsequent processing of all samples into cDNA in a single batch. cDNA was prepared using Superscript IV VILO master mix (ThermoFisher) and variable volumes of RNA depending on the desired final concentration. Final concentrations of cDNA were calculated to be 250ng per sample for PBMCs, 75ng per sample for CD19^+^, 200ng per sample for CD3^+^, and 250ng per sample for RPMI-8226 myeloma cell positive controls. The cDNA synthesis was carried out using a Thermal Cycler (Bio-Rad) with the heat cycling set as directed by the cDNA synthesis kit instructions. The cDNA samples were stored at −20 °C until ready to be used for qRT-PCR.

### Quantitative real time PCR (qRT-PCR) setup

qRT-PCR reactions were prepared using Taqman Fast Advanced Master Mix (ThermoFisher), RNase-free water (ThermoFisher), 1 μL of cDNA input per reaction, and multiplexed Taqman primer/probe sets (10 μL reaction volume). qRT-PCR analysis was performed on a QuantStudio3 instrument (ThermoFisher) following the protocol and heat cycling parameters outlined by the manufacturer. The primers used in qPCR measurements are shown in Table 2.

**Table 2:**
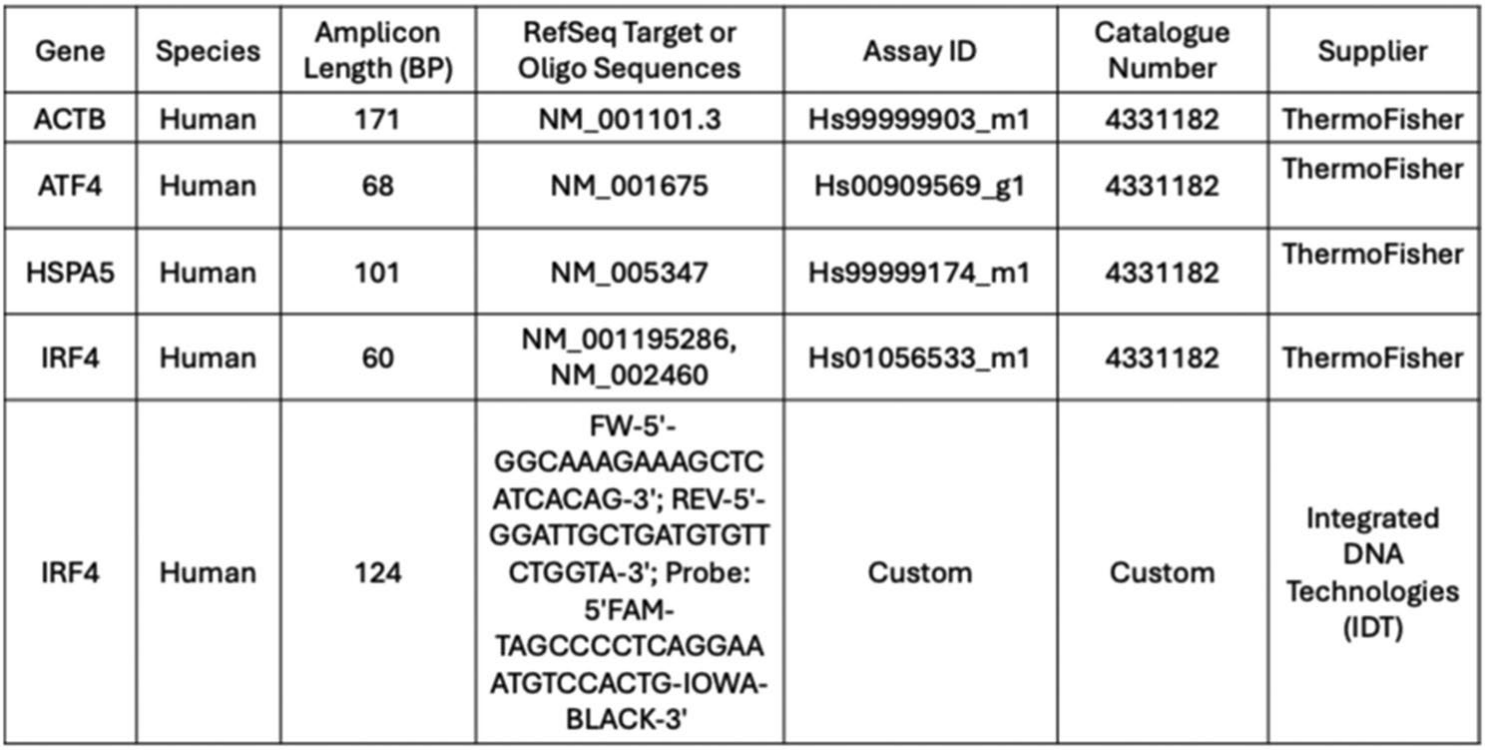
Gene targets and primer/probe assays used for qRT-PCR analyses

### Flow cytometry

For flow cytometry analyses, a peripheral blood panel was used which had been previously validated on normal cryopreserved PBMC aliquots [4]. Technical duplicates from each sample were run to ensure accuracy of the resulting data (5 patients, 3 tube types and 2 timepoints each; 60 samples in total, including replicates). Stability of cell surface antigens and frequency of positive cells were analyzed for the following cell fractions: live cells, CD3+, CD19+, and CD14+. Along with this, IRF4 median fluorescence intensity (MFI) was analyzed in CD3+, CD19+, and CD14+ fractions. The antibodies used in the flow cytometry analysis are shown in Table 3.

**Table 3:**
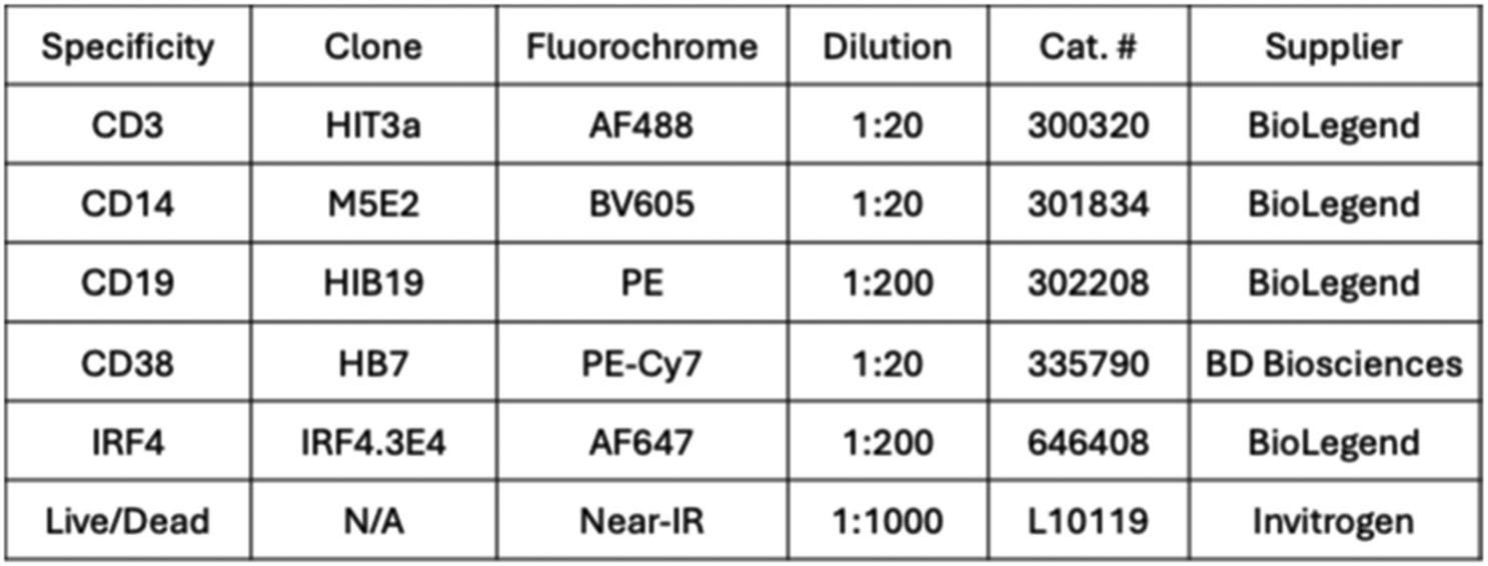
Antibodies used in flow cytometry analyses for IRF4 biomarker detection

### Statistical analyses and data availability

Data were analyzed and visualized using Microsoft Excel and GraphPad Prism (Prism 10, GraphPad Software). All data were subjected to appropriate analytical tests to determine statistically significant differences between conditions and timepoints (one-way ANOVA with post-hoc Tukey-Kramer), however since this study was designed to compare different technical variables rather than biological conditions, there is no absolute control available for comparison. For this reason, statistical differences were not annotated in the figures at this time but can be made available upon request. In addition, no genomics or transcriptomics data or linked phenotype and genotype data were generated in this study. All relevant raw data will be made available for non-commercial purposes upon request.

## Results

### Sample processing and study design

The first goal of this study was to determine the optimal technical and analytical conditions that would enable stable detection of IRF4 as a valuable disease-associated molecular biomarker for human conditions typified by aberrant IRF4 expression, such as MM. An optimization cohort was obtained, comprised of freshly drawn whole blood samples purchased from a commercial supplier (AllCells). Samples from a total of 5 healthy donors were requested. All donors were non-smokers, and the general demographic information provided by the supplier is presented in Table 1. We specifically chose to focus our studies on samples that were subjected to a 24- or 48-hour delay in sample processing as this would be the expected interval between blood sample collection and processing in most laboratories for samples collected in multi-site clinical trials across the continental United States. Because one primary endpoint was to evaluate RNA and protein stability and enable isolation of subpopulations of the mononuclear cell fraction (B and T cells), we opted to compare blood collection tubes of the following 3 types: EDTA (10 mL tubes) Paxgene ccfDNA (8.5 mL tubes), and Na Citrate (4.5 mL tubes). The overall experimental design and workflow are depicted in Fig. 1A.

In comparing the sample integrity across these three tube types tested at the 24 hr mark, the least amount of hemolysis was observed, and the highest volume of plasma was recovered in Na Citrate tubes under standard processing conditions (centrifugation of whole blood at 200xg for 10 mins), indicating that plasma separation was the best overall in this tube type (Supplemental Fig. 1A). However, it is important to note a potential minor limitation of this comparison is that Na Citrate tubes contain a liquid additive (approximately 1 part additive to 9 parts whole blood), which minimally dilutes plasma compared to spray-coated tubes. The amount of hemolysis and plasma separation was comparable in EDTA tubes to Na Citrate tubes, but with slightly increased hemolysis and poorer separation observed in the EDTA tubes. In Paxgene ccfDNA tubes, plasma was almost non-recoverable under standard plasma processing conditions, so the samples received in shipment 2 were processed according to alternative Paxgene manufacturer protocols (centrifugation at 1600xg for 15 mins) for Paxgene tubes only. Plasma recovery with the Paxgene protocol was substantially improved, with higher total volumes of plasma recovered in samples from shipment 2. However, just as with the Na Citrate tubes, the Paxgene tubes contain a liquid additive, approximately 1.5 parts additive to 8.5 parts whole blood, which modestly dilutes the plasma.

At the 48 hr mark, observable differences in hemolysis and plasma separation between tube types were much less stark. Substantially more hemolysis was observed in all tube types, and as a result plasma separation was decreased in all tube types overall. However, the previous trends held true in that Na Citrate tubes exhibited the least hemolysis and plasma separation, followed closely by EDTA tubes, and then Paxgene tubes.

### Longitudinal evaluation of cell viability and yield

After in-lab processing of the specimens, the live-cell count of each specimen was determined. For samples processed 24 hours after being shipped, raw live cell counts for each sample varied somewhat in proportion to the tube collection volumes (Fig. 1B–D), since standard EDTA collection tubes contain a larger volume of blood. After adjusting the live cell counts for the total blood volume (in mL) collected in each tube type, EDTA and Na Citrate tubes contained roughly similar average numbers of cells/mL, while Paxgene tubes produced the lowest cell counts (Fig. 1C). For samples processed in the 48-hour group, the trends remained similar to those in the 24-hour group, though the adjusted cell counts/mL were slightly higher on average (Fig. 1B). Overall, after normalizing the cell counts to the volume in each tube at each time point, EDTA and Na Citrate tubes contained similar average numbers of cells/mL, and Paxgene tubes exhibited the lowest relative viable cell counts (Fig. 1C).

### RNA yield and evaluation of IRF4 mRNA stability

**Fig. 2:**
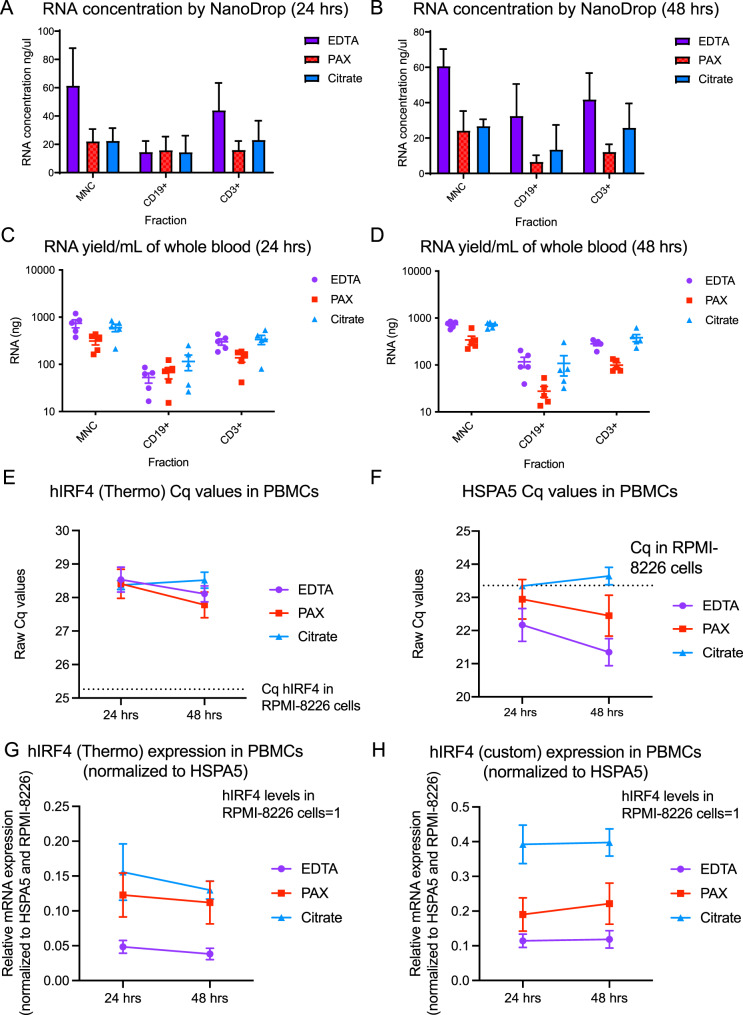
RNA concentrations, total RNA yields, and qRT-PCR measurements in healthy donor PBMCs. (**A**, **B**) Average RNA concentrations (ng/uL) in each tube type (EDTA, Na Citrate, PAXgene) at 24 hrs (**A**) and 48 hrs (**B**) measured by Nanodrop in *n* = 5 individual donors, grouped by cell fraction (MNC, CD3+, CD19+). (**C**, **D**) Average RNA yield/mL (adjusted to total tube volume) at 24 hrs (**C**) and 48 hrs (**D**) in each tube type (EDTA, Na Citrate, PAXgene) measured by Nanodrop in *n* = 5 individual donors, grouped by cell fraction (MNC, CD3+, CD19+). (**E**, **F**) Average hIRF4 (thermo) (**E**) and HSPA5 (thermo) (**F**) Cq values measured by qRT-PCR in the isolated PBMCs of *n* = 5 individual donors in each tube type (EDTA, Na Citrate, PAXgene) grouped by 24 and 48 hr processing intervals. Dashed lines represent mean Cq values of hIRF4 (**E**) and HSPA5 (**F**) in RPMI-8226 cells shown for comparison. (**G**, **H**) Relative mRNA expression of hIRF4 (thermo Taqman assay, **G** and custom Taqman assay, **H**), normalized to HSPA5 (thermo) as a housekeeping gene, in the isolated PBMCs of *n* = 5 individual donors in each tube type (EDTA, Na Citrate, PAXgene) grouped by 24 and 48 hr processing intervals

Next, we evaluated total and relative RNA yields at each time point and for each tube type. In both the 24 hr and 48 hr processing groups, the highest RNA concentration was obtained from EDTA tubes, followed by Na Citrate tubes, and then Paxgene tubes (Fig. 2A, B). As for the live cell counts, this result was likely due to the volume of blood (10 mL tubes) collected in the EDTA tubes. Adjusting the RNA yield to the volume of each tube (calculated per mL to normalize for different whole blood volumes in each tube type) showed that the Na Citrate tubes had the greatest RNA yield, followed closely by the EDTA tubes, with Paxgene tubes showing the lowest total RNA yield/mL over both time points (Fig. 2C and D). RNA yields for all cell fractions were roughly similar in EDTA and Na Citrate tubes, with slightly higher average B cell RNA yields obtained from EDTA tubes at the 48 hr timepoint (Fig. 2C and D). In contrast, the total and relative RNA yields for Paxgene tubes were relatively low for all donor samples received in both shipments at both timepoints tested, even with processing using the Paxgene recommended protocol (Fig. 2A–D).

### qPCR quality control and optimal housekeeping gene selection

After the whole blood processing, cell fraction isolation, and RNA extraction of each specimen, cDNA was prepared in order to evaluate the stability of human (h)IRF4 mRNA levels via qRT-PCR, and select a stable housekeeping gene for analysis of real-world samples with variable post-collection handling conditions. First, the range and standard deviations of quantification cycle (Cq) values from the qRT-PCR assays were analyzed as a quality control measure of the technical replicates evaluated. With a cDNA equivalent of 12.5ng of RNA input per reaction, for all PBMC samples analyzed, the Cq values ranged from 26 to 28 for hIRF4 and from 20 to 23 for β-actin (*ACTB*), the well-established housekeeping gene that was initially used to normalize qRT-PCR outputs (Supplemental Fig. 1B). We observed that hIRF4 expression was detectable in all cell fractions (PBMCs, CD19+ B cells and CD3+ T cells) and tube types tested (Fig. 2E, Supplemental Fig. 1C). We also compared the relative hIRF4 Cq values and expression in each isolated cell fraction to an established MM cell line, RPMI-8226. RPMI-8226 cells were found to have the highest transcript abundance (lowest Cq values, Fig. 2E), followed by the isolated CD19 cells, and finally the total PBMC fraction and the CD3 fraction which had similarly low levels at 24 hours (Supplemental Fig. 1C–E). When using *ACTB* as the reference gene, we were surprised to observe that relative hIRF4 expression in PBMCs appeared to increase by a 2.2-to-3.4-fold change in all tube types after a 48 hr delay in processing, with similar trends seen in the CD19+ and CD3+ fractions (Supplemental Fig. 1C–E). Upon further evaluation, we determined that this may reflect a technical artefact related to changes in the absolute Cq values of *hIRF4* (decreasing at 48hrs compared with 24hrs) in EDTA and Paxgene tubes (Fig. 2E), while the *ACTB* Cq values in the same samples increased in Na Citrate and Paxgene tubes over the same interval (Supplemental Fig. 1F). Notably, absolute Cq values of *hIRF4* were stable at 24 and 48hrs in samples collected in Na Citrate tubes, and this was confirmed using an alternate custom primer/probe set (Fig. 2E, Supplemental Fig. 1G). Thus, we analyzed alternative reference genes (*ATF4* and *HSPA5*) and found that the Cq values for both of these gene products were relatively stable across both time points analyzed (Fig. 2F, Supplemental Fig. 1H). We subsequently identified literature reports linking ATF4 with IRF4 function and gene expression programs [10, 11] and therefore we excluded *ATF4* from further consideration. Together, Na Citrate tubes along with the housekeeping gene *HSPA5* were selected as the most consistent parameters that enabled stable qRT-PCR-based quantification of *hIRF4* under variable pre-analytical conditions (Fig. 2G and H).

### Evaluation of IRF4 protein stability across immune cell subpopulations

**Fig. 3:**
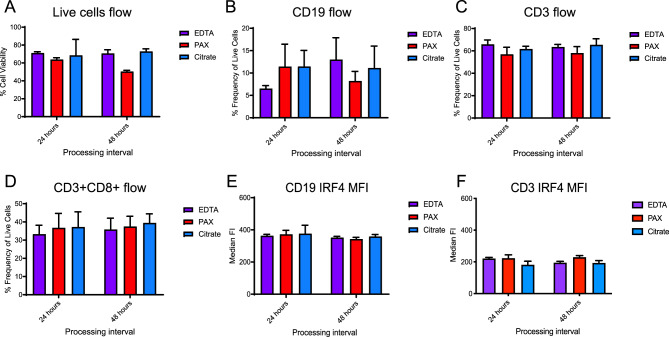
Flow cytometry (FC) analysis of immune cell frequencies and IRF4 median fluorescence intensity (MFI). (**A**-**D**) Average percent cell viability (**A**) and percent frequency of live CD19+ (**B**), CD3+ (**C**), and CD3+CD8+ (**D**) cells from *n* = 5 individual donors in each tube type (EDTA, Na Citrate, PAXgene) determined by FC and grouped by 24 and 48 hr processing intervals. (**E**, **F**) Average IRF4 MFI in live CD19+ (**E**) and CD3+ (**F**) cells from *n* = 5 individual donors in each tube type (EDTA, Na Citrate, PAXgene) determined by FC and grouped by 24 and 48 hr processing intervals

Next, the stability and relative abundance of cell surface and intracellular antigens were investigated via flow cytometry across tube types and timepoints. Overall live cell recovery, determined via Near-IR staining, was similar in EDTA and Na Citrate tubes, but slightly lower in Paxgene tubes, especially at the 48-hour mark (Fig. 3A). The frequency of CD19+ B lymphocytes (5–10% of live single cells) and CD14+ monocytes (2–10% of live single cells) varied somewhat between tube types but showed the least variation between 24 and 48 hrs in Na Citrate tubes (Fig. 3 B and Supplemental Fig. 1I). Of the isolated cell fractions, both EDTA and Na Citrate tubes also showed stable frequencies of CD3+ cells at levels greater than 60%, with 30–40% of these cells also being CD8+ (Fig. 3 C and D). Together, the relative abundance of most immune cell subtypes remained the most consistent across 24 and 48 hr processing delays in Na Citrate tubes (Fig. 3A–D).

Further investigation via flow cytometry was done to evaluate the mean fluorescence intensity (MFI) of IRF4 and therefore the relative abundance and stability of the IRF4 protein expressed within each isolated cell fraction. IRF4 protein expression was detectable in all PBMC subsets analyzed, with expression being the highest in CD14+ cells, followed by CD19+, and then CD3+ respectively (Fig. 3 E and F, Supplemental Fig. 1J and K). The technical replicates were highly consistent (e.g. standard deviations averaged < 5% for CD19+ IRF4 MFI values across replicates for all conditions).

In evaluating differences between conditions, IRF4 protein levels in all immune cell sub-populations were remarkably stable across tube types and 24- and 48-hour processing intervals. Together, IRF4 protein biomarker detection by flow cytometry exhibits excellent stability in primary blood samples collected across a variety of pre-analytical conditions, including up to 48 hours of pre-analytical processing delays (Fig. 3).

## Discussion

Existing literature has established that most whole blood analytes, including RNA and immune cell surface markers, are stable under normal shipping conditions (4–25°C) and with short-term processing delays ( < 15 hours) [12, 13]. Along with this, previous studies have established conditions offering relative stability of specific whole blood analytes in unfractionated samples up to 7 days through a variety of different means, such as fixation, and different tube types including Paxgene, Tempus, EDTA, and Cyto-Chex [14–16]. However, most of these studies only compare 2 differing tube types and have a single endpoint, commonly flow cytometry, microarray analysis, or bulk sequencing. In order to obtain a more holistic overview of the most optimal processing conditions specifically relevant to the quantification of a key MM biomarker, IRF4, we tested 3 different tube types (EDTA, Paxgene ccfDNA, and Na Citrate) over 2 different time points (24- and 48-hours after sample collection) with two different analytical endpoints (qRT-PCR and flow cytometry). We also considered using heparin tubes, however since this anticoagulant may interfere with downstream PCR applications [17, 18], we excluded it from our conditions for this study. We also evaluated multiple cell fractions representing discrete immune subpopulations, PBMCs, CD3+, CD19+, and CD14+, to produce detailed recommendations for more specialized future research studies.

In rigorous qRT-PCR studies, we found that hIRF4 expression was detectable in all cell fractions (PBMCs, CD19+ B cells and CD3+ T cells) and tube types tested (Na Citrate, EDTA, Paxgene ccfDNA). Notably, raw Cq values of hIRF4 mRNA remained constant at 24 and 48hrs post-collection in PBMCs isolated from Na Citrate tubes. On the other hand, raw Cq values of *ACTB* tended to increase over time in some tube types (Na Citrate and EDTA). Processing delays of 48hrs may result in an increase in IRF4 mRNA expression in some tube types and a decrease in *ACTB* transcripts as a result of potential degradation/destabilization of mRNA transcripts over time. An increase in IRF4 mRNA expression may reflect biological immune cell responses to cellular stress, potentially through the increased release of IL-4 and/or stimulation via CD40, potentially counteracting the degradation which causes the notable decrease in *ACTB* transcripts [19, 20].

Based on flow cytometry analyses of PBMC cell subsets, Na Citrate tubes exhibited the highest stability of cell surface antigens across 24–48-hour processing delays. Furthermore, relative frequencies of CD19+ and CD14+ cells were the most stable in Na Citrate tubes across processing time points. Intranuclear IRF4 protein levels were remarkably stable in all cell populations tested across all tube types, with the highest relative expression in CD14+ and CD19+ fractions, and lowest in CD3+ fractions, in this cohort of healthy donors. The flow cytometry results were also consistent with qRT-PCR results showing highest expression of IRF4 mRNA in CD19+ fractions and lower in CD3+ fractions (CD14+ fractions were not tested by qRT-PCR due to the focus on lymphoid populations for IRF4 detection in this study).

Overall, Na Citrate tubes provided superior plasma recovery and hIRF4 transcript stability, with suitable total MNC and RNA yields obtained even in the setting of lower volumes of whole blood input (4.5 mL in Na Citrate versus 10 mL in EDTA tubes). Housekeeping gene analyses demonstrated that Na Citrate tubes also provided superior *HSPA5* and *ATF4* transcript stability compared to other tube types, however we determined that *ATF4* may not be an optimal reference gene for studies of IRF4 due to potential regulatory control of ATF4 by IRF4 [11].

## Conclusions

Among the conditions tested in this biomarker detection study, Na Citrate tubes were determined to be the optimal tube type for the collection of blood samples for stable detection of IRF4 mRNA or protein. Due to the liquid additive included in Na Citrate tubes, these are less optimal for analysis of plasma or serum analytes, and a separate sample collected in a serum-separator tube would be recommended for such endpoints. Furthermore, shipment of blood samples at room temperature and with a delivery timeline within 48 hours of sample collection should maintain analyte stability under these conditions. Overall, Na Citrate tubes maintained the stability of IRF4 mRNA and protein expression even under conditions of tube processing delays. EDTA tubes performed similarly for IRF4 protein analyses, but the same result was not seen in IRF4 mRNA analyses. Furthermore, IRF4 protein appears to be slightly more stable than mRNA after variable processing delays, but both analytes are expected to be detectable in clinical samples, thus enabling reliable analysis of clinical samples collected from multiple sites and shipped to a central laboratory. While these recommended conditions represent an optimal guideline for detection of IRF4 as a biomarker in human clinical blood samples, these findings may also inform future validation and clinical trial correlative studies involving IRF4 quantification in other tissue types (e.g., bone marrow) [21, 22].

## Electronic supplementary material

Below is the link to the electronic supplementary material.

Supplementary material 1

## Data Availability

Data are presented within the figures and tables. No new materials or resources were generated in the course of this study as all materials were used in the analyses performed.

## Abbreviations

ACTB: β-Actin
ASO: Antisense Oligonucleotide
ATF4: Activating Transcription Factor 4
BM: Bone Marrow
Cq: Quantification Cycle
EDTA: Ethylenediaminetetraacetic acid
HSPA5: Heat Shock Protein Family A Member 5
IRF4: Interferon-Regulatory Factor 4
MFI: Median Fluorescence Intensity
MM: Multiple Myeloma
PB: Peripheral Blood
qRT-PCR: Quantitative Real-Time PCR
