# Supplementary material for: Optimal conditions for quantification of IRF4 mRNA and protein as a correlative biomarker in clinical cancer studies
Source: J Transl Med. 2026 Mar 16;24:575. doi: 10.1186/s12967-026-07803-0 (PMC13104480; doi:10.1186/s12967-026-07803-0)

Supplemental Figure 1: Quality assessments and qRT-PCR and flow cytometry measurements in healthy donor PBMCs. (**A**) Photograph of EDTA, Na Citrate, and PAXgene tubes (from left to right) after being gently rocked for 24 hrs (after delivery) and centrifuged at 200xg for 10 minutes. (**B**) Scatter plot of quality control (QC) assessments of average Ct/Cq values for *ACTB* and *hIRF4* (x-axis) versus the corresponding standard deviations (y-axis) of two technical replicates analyzed per individual sample for each gene displayed. (**C**-**E**) Relative *hIRF4* (Thermo) mRNA expression, normalized to an *ACTB* (Thermo) housekeeping gene, in PBMCs (C) and CD19+ (D) and CD3+ (E) fractions from n=5 individual donors for each tube type (EDTA, Na Citrate, PAXgene) grouped by 24 and 48 hr processing intervals. (**F**-**H**) Average *ACTB* (Thermo) (F), *hIRF4* (custom) (G), and *ATF4* (Thermo) (H) Cq values in PBMCs from n=5 individual donors for each tube type (EDTA, Na Citrate, PAXgene) grouped by 24 and 48 hr processing intervals. Dashed lines represent the mean Cq value of *ACTB* (F), *hIRF4* (G), and *ATF4* (H) in RPMI-8226 cells shown for comparison. (**I**) Percent live CD14+ cells from n=5 individual donors for each tube type (EDTA, Na Citrate, PAXgene) determined by flow cytometry and grouped by 24 and 48 hr processing intervals. (**J**, **K**) Relative IRF4 MFI in live CD14+ (J) and CD3+CD8+ (K) cells from n=5 individual donors for each tube type (EDTA, Na Citrate, PAXgene) determined by flow cytometry and grouped by 24 and 48 hr processing intervals.

Supplemental Figure 1

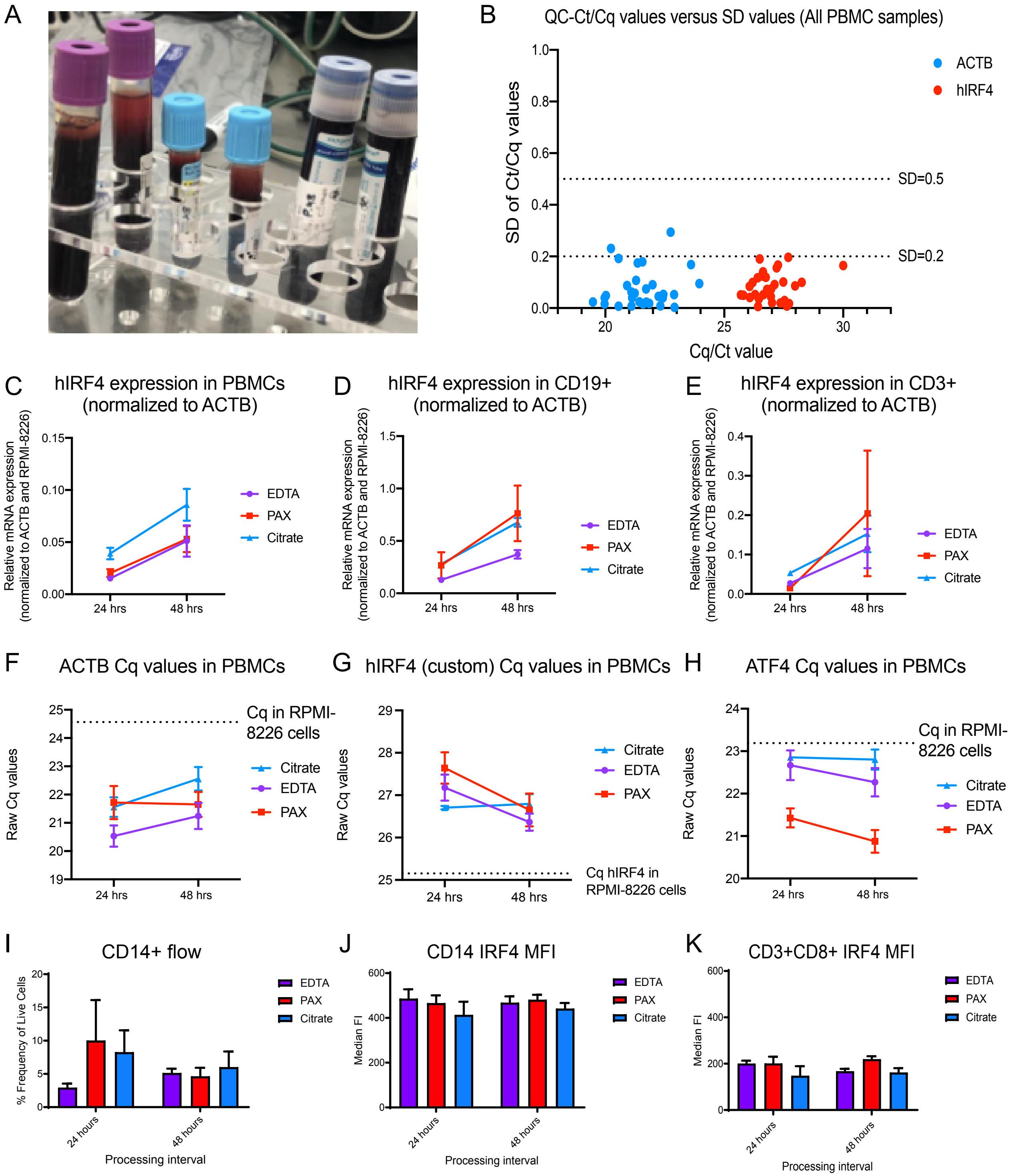

Supplement: Supplementary file 1 — Supplementary material 1 [file 12967_2026_7803_MOESM1_ESM.pdf]
